# Approaches to interventional fluoroscopic dose curves

**DOI:** 10.1120/jacmp.v17i1.5788

**Published:** 2016-01-08

**Authors:** Kevin A. Wunderle, Joseph T. Rakowski, Frank F. Dong

**Affiliations:** ^1^ Department of Radiology Cleveland Clinic Cleveland OH; ^2^ Department of Radiation Oncology Wayne State University School of Medicine Detroit MI USA

**Keywords:** fluoroscopy, ADRC, dose curves

## Abstract

Modern fluoroscopes used for image‐based guidance in interventional procedures are complex X‐ray machines, with advanced image acquisition and processing systems capable of automatically controlling numerous parameters based on defined protocol settings. This study evaluated and compared approaches to technique factor modulation and air kerma rates in response to simulated patient thickness variations for four state‐of‐the‐art and one previous‐generation interventional fluoroscopes. A polymethyl methacrylate (PMMA) phantom was used as a tissue surrogate for the purposes of determining fluoroscopic reference plane air kerma rates, kVp, mA, and variable copper filter thickness over a wide range of simulated tissue thicknesses. Data were acquired for each fluoroscopic and acquisition dose curve within each vendor's default abdomen or body imaging protocol. The data obtained indicated vendor‐ and model‐specific variations in the approach to technique factor modulation and reference plane air kerma rates across a range of tissue thicknesses. However, in the imaging protocol evaluated, all of the state‐of‐the‐art systems had relatively low air kerma rates in the fluoroscopic low‐dose imaging mode as compared to the previous‐generation unit. Each of the newest‐generation systems also employ Cu filtration within the selected protocol in the acquisition mode of imaging; this is a substantial benefit, reducing the skin entrance dose to the patient in the highest dose‐rate mode of fluoroscope operation. Some vendors have also enhanced the radiation output capabilities of their fluoroscopes which, under specific conditions, may be beneficial; however, these increased output capabilities also have the potential to lead to unnecessarily high dose rates. Understanding how fluoroscopic technique factors are modulated provides insight into the vendor‐specific image acquisition approach and may provide opportunities to optimize the imaging protocols for clinical practice.

PACS number: 87.59.C‐

## I. INTRODUCTION

Modern fluoroscopes used for real‐time, image‐based guidance in interventional procedures are complex X‐ray machines, with advanced image acquisition and processing systems capable of automatically controlling numerous parameters based on defined protocol settings.^(1)^ Advances in X‐ray generation have allowed for the production of nearly constant applied voltage and X‐ray tubes capable of greater radiation output; this is evident with the newest generation of X‐ray tubes used in interventional fluoroscopes from GE (Waukesha, WI) and Siemens (Erlangen, Germany), which have 100 kVp power ratings of up to 100 kW and 90 kW, respectively.^(2)^ These advances in X‐ray generation, combined with advances, such as cone‐beam computed tomography (CBCT) and 3D fluoroscopic road‐mapping, have facilitated the expansion of the vascular interventional clinical repertoire to include diseases and disease states previously only treatable with surgery.^(3)^ Although these fluoroscopically guided interventional (FGI) procedures are generally safer and offer outcomes similar to, or better than, their surgical alternatives, many are capable of inducing radiogenic tissue reactions.^(3)^ To limit the risk of tissue reactions, other technological advances, such as pulsed only X‐ray beams, variable copper filtration to lower skin entrance dose and potentially preserve contrast by allowing for lower kVps, and automatic dose rate control (ADRC) have been implemented and refined over the last 20 years.^(4)^

The purpose of this study was to evaluate and compare dose rate curves and approaches to technique factor modulation, controlled by the ADRC, in response to simulated patient thickness variations for several state‐of‐the‐art fluoroscope models. The ADRC is the vendor‐specific, software‐based operational logic that controls the X‐ray generation system and associated parameters, such as kVp, mA, ms, and copper filtration thickness. In general, the goal of the ADRC is to maintain a specified radiation dose to the image receptor, within regulatory or X‐ray tube power limitations, based on the imaging protocol chosen by the operator. As stated by the American Association of Physicists in Medicine (APPM) Task Group 125 (TG‐125), knowledge of the operational logic driving ADRC for fluoroscopic systems is essential to assess whether the units are functioning properly; proper functioning affects both image quality and patient radiation dose.^(5)^ Understanding how the fluoroscopic technique factors are modulated also provides knowledge of vendor‐specific image acquisition approaches, which may provide insight into opportunities for optimization based on the clinical procedure's or operator's imaging requirements.

The operational logic of fluoroscopes incorporating ADRC has been investigated for various generations of fluoroscopic equipment over the last three decades.^1^, ^4^, ^5^, ^6^, ^7^ Increases in computing power and speed have advanced these capabilities to include numerous parameters on the image acquisition and processing systems.^(1)^ The AAPM TG‐125 report defined and summarized existing fluoroscopic data, and the current work attempts to add information regarding newer‐generation fluoroscopic approaches to this knowledge base.

For the purpose of this study, imaging protocols shall refer to the selected examination set on the fluoroscope workstation that specifies the parameters used for X‐ray generation and image processing. Vendor‐default protocols were evaluated in this study, which generally represent a starting point for clinically used protocols. Imaging protocols should be optimized for the particular clinical task; procedures requiring high spatial resolution or visualization of subtle differences in contrast may necessitate higher radiation dose rates, whereas the clinical requirements for other procedures may allow for reduced radiation dose rates.^(8)^ Any modifications to the imaging protocols must be done in consultation with the clinical team and with a proficient understanding of the vendor‐specific imaging protocols and parameters.

## II. MATERIALS AND METHODS

Four state‐of‐the‐art and one previous‐generation interventional C‐arm fluoroscopes (still on the market) from three manufacturers were evaluated, including a GE Discovery IGS 730 (Waukesha, WI), Philips Allura FD 20 with Clarity (Best, Netherlands), and Siemens Artis Q, Artis Q.zen and Artis Zee systems (Erlangen, Germany). All testing was performed using the vendors' default abdomen or body imaging protocols, with the exception of the GE system which does not provide default organ‐based programs. GE offers the choice of several default dose curves that can be selected and applied to clinical protocols, affecting both the fluoroscopic and acquisition modes of imaging. For the GE unit, the dose curves chosen were the ones clinically used and representative of a probable default abdomen protocol.

All ${\dot{K}}_{a,r}$ (air kerma rate at the interventional reference point [IRP]) values reported in this study were determined and displayed by each of the fluoroscopes during irradiation. With the exception of the GE Discovery unit, all ${\dot{K}}_{a,r}$ values were measured by a Kerma‐Area‐Product meter (KAP‐meter) integrated into the fluoroscope assembly to monitor X‐ray tube output. The GE Discovery used factory preprogrammed look‐up tables (LUTs), based on the system geometry and technique factors, to determine the KAP (the product of air kerma and the X‐ray beam field size on the same plane as the measurement of the air kerma), $K_{\text{ar}}$ (air kerma at the IRP) and ${\dot{K}}_{a,r}$. Accuracy of the displayed $K_{\text{ar}}$ for all fluoroscopes was determined by comparing displayed values to measurements made with a calibrated Radcal Accu‐Pro dosimeter (Monrovia, CA) with a Radcal 10x6‐6 ionization chamber placed free‐in‐air at the IRP (the point [or plane] in space where the $K_{\text{ar}}$ is calculated; the International Electrotechnical Commission definition is 15 cm toward the X‐ray tube from the isocenter of the C‐arm gantry, all fluoroscopes tested use this definition). Because the allowed deviation of the displayed $K_{\text{ar}}$ and KAP, per the IEC and FDA, is ±35%, correction factors (CFs) were determined using the external ionization chamber as a reference. With lead in the beam, measurements were made to determine CFs (Table 1) for the fluoroscopic and acquisition modes of operation in the chosen imaging protocol, which were then applied to their respective data:

(1) $$CF=\frac{K_{a,r,ex}}{K_{a,r,in}}$$

where $K_{a,r,ex}$ is the cumulative air kerma at the IRP as measured with the external calibrated ionization chamber, and $K_{a,r,in}$ is the cumulative air kerma at the IRP as determined with the integ rated KAP‐meter or LUT.

For this study, all reported ${\dot{K}}_{a,r}$ values were those reported at the IRP for each fluoroscope. However, because of geometric differences between the fluoroscope gantries, the focal spot to IRP distances, the floor‐to‐focal‐spot distances, and the floor‐to‐IRP distances are different among the fluoroscopes evaluated (Table 2). However, if the assumption is made, regarding fluoroscope geometry, that most clinical imaging will be performed at an operator‐preferred table height (floor‐to‐procedure‐table height), these geometric differences result in a relatively small deviation in ${\dot{K}}_{a,r}$ when the procedure table is placed at the respective IRP. If the ${\dot{K}}_{a,r}$ from the largest floor to table height is adjusted to match that of the smallest floor to table height, the deviation is approximately 5% (see Appendix A for further explanation of this deviation). Therefore, the ${\dot{K}}_{a,r}$ from each fluoroscope was used without geometric normalizing.

**Table 1:** Calculated correction factors applied to the displayed air kerma rates.

*Vendor Make and Model*	*Correction Factor for Fluoroscopic Mode*	*Correction Factor for Acquisition Mode*
GE Discovery IGS	1.03	1.09
Philips Allura with Clarity	1.16	1.16
Siemens Artis Q	1.13	1.06
Siemens Artis Q.zen	1.03	1.09
Siemens Artis Zee	1.21	1.12

**Table 2:** Fluoroscope reference distances.

*Vendor Make and Model*	*Interventional Reference Point (cm distance from focal spot)*	*Floor to Focal Spot Distance (cm)*	*Reference Point Distance From Floor (cm)*
GE Discovery IGS	67	25	92
Philips Allura w/ Clarity	61.5	29.5	91
Siemens Artis Q	63.5	30.5	93.5
Siemens Artis Q.zen	60	30	90
Siemens Artis Zee	63.5	31	94.5

For each fluoroscope, with the table pad removed, the surface of the procedure table was placed at the respective IRP with 35.56 cm (14 inches) of polymethyl methacrylate (PMMA) placed on top. The image receptor was lowered as close as possible to the PMMA. The X‐ray field of view closest to 40 cm was used for all measurements, with the manufacturers' standard antiscatter grid in place. The ${\dot{K}}_{a,r}$, X‐ray tube potential (kVp), X‐ray tube current (mA), and copper filtration (mm Cu) were recorded for each PMMA thickness evaluated. Measurements were made in the vendors' default abdomen or body protocols for a 4 frames per s (fps) acquisition irradiation and 15 pulses per s (pps) fluoroscopic irradiations in the low‐, normal‐ and high‐dose modes, as applicable. This process was repeated for PMMA thicknesses ranging from 2.54 to 35.56 cm in 2.54 cm increments, while maintaining the initial SID (source‐to‐image distance) and OID (object‐to‐image distance).

## III. RESULTS

**Figure 1:**
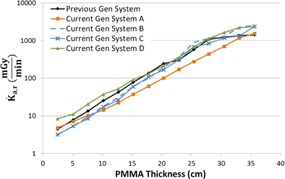
${\dot{K}}_{a,r}$ vs. PMMA phantom thickness for a 4 fps acquisition.

**Figure 2:**
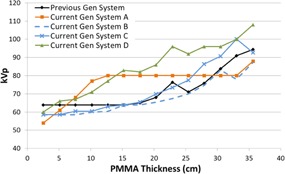
kVp vs. PMMA phantom thickness for a 4 fps acquisition.

**Figure 3:**
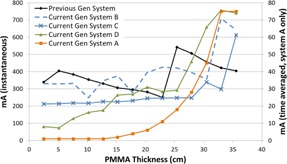
mA vs. PMMA phantom thickness for a 4 fps acquisition.

**Figure 4:**
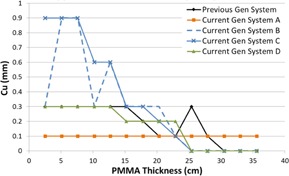
Cu thickness vs. PMMA phantom thickness for a 4 fps acquisition.

1, 4 were generated with data from the vendors' default abdomen or body protocols in a 4 fps acquisition. Figure 1 illustrates ${\dot{K}}_{a,r}$ with respect to phantom thickness, with thicknesses ranging from 2.54 to 35.56. For this same acquisition protocol, variation of the X‐ray tube potential with phantom thickness is shown in Fig. 2, variation of tube current with phantom thickness is shown in Fig. 3, and variation of the copper filter thickness with phantom thickness is shown in Fig. 4. Note that some vendors choose to report a time‐averaged X‐ray tube current, whereas others report a maximum instantaneous value; both are represented in the two‐axes mA figures.

**Figure 5:**
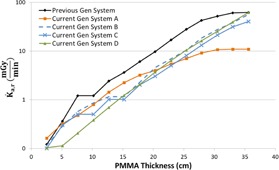
${\dot{K}}_{a,r}$ vs. PMMA phantom thickness in the fluoroscopic low‐dose mode at 15 pps.

**Figure 6:**
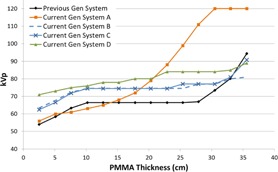
kVp vs. PMMA phantom thickness in the fluoroscopic low‐dose mode at 15 pps.

**Figure 7:**
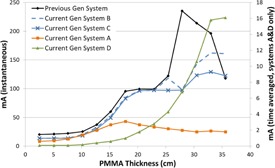
mA vs. PMMA phantom thickness in the fluoroscopic low‐dose mode at 15 pps.

**Figure 8:**
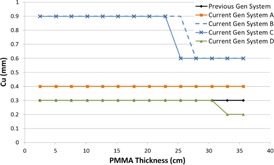
Cu thickness vs. PMMA phantom thickness in the fluoroscopic low‐dose mode at 15 pps.

Data used to generate 5, 8 were acquired with the vendors' default abdomen or body protocols in a 15 pps fluoroscopic low‐dose mode. Figure 5 illustrates ${\dot{K}}_{a,r}$ with respect to phantom thickness, with thicknesses ranging from 2.54 to 35.56 cm. For this same fluoroscopic protocol, variation of the X‐ray tube potential with phantom thickness is shown in Fig. 6, variation of tube current with phantom thickness is shown in Fig. 7, and variation of the copper filter thickness with phantom thickness is shown in Fig. 8.

**Figure 9:**
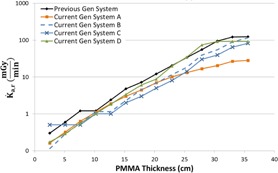
${\dot{K}}_{a,r}$ vs. PMMA phantom thickness in the fluoroscopic normal‐dose mode at 15 pps.

**Figure 10:**
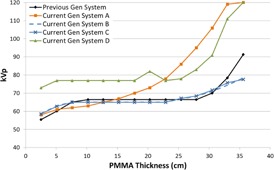
kVp vs. PMMA phantom thickness in the fluoroscopic normal‐dose mode at 15 pps.

**Figure 11:**
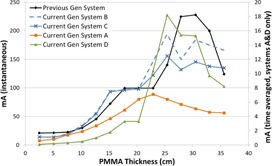
mA vs. PMMA phantom thickness in the fluoroscopic normal‐dose mode at 15 pps.

**Figure 12:**
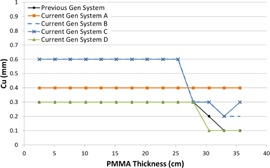
Cu thickness vs. PMMA phantom thickness in the fluoroscopic normal‐dose mode at 15 pps.

Data used to generate 9, 12 were acquired with the vendors' default abdomen or body protocols in a 15 pps fluoroscopic normal dose mode. Figure 9 illustrates ${\dot{K}}_{a,r}$ with respect to phantom thickness, with thicknesses ranging from 2.54 to 35.56 cm. For this same fluoroscopic protocol, variation of the X‐ray tube potential with phantom thickness is shown in Fig. 10, variation of the mA with phantom thickness is shown in Fig. 11, and variation of the copper filter thickness with phantom thickness is shown in Fig. 12.

**Figure 13:**
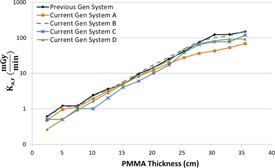
${\dot{K}}_{a,r}$ for PMMA phantom thickness in the fluoroscopic high‐dose mode at 15 pps.

**Figure 14:**
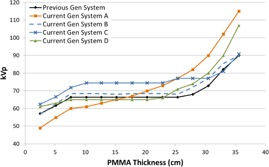
kVp vs. PMMA phantom thickness in the fluoroscopic high‐dose mode at 15 pps.

**Figure 15:**
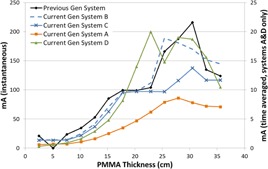
mA vs. PMMA phantom thickness in the fluoroscopic high‐dose mode at 15 pps.

**Figure 16:**
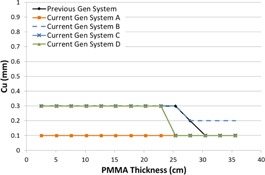
Cu thickness vs. PMMA phantom thickness in the fluoroscopic high‐dose mode at 15 pps.

Data used to generate 13, 16 were acquired with the vendors' default abdomen or body protocols in a 15 pps fluoroscopic high‐dose mode. Figure 13 illustrates ${\dot{K}}_{a,r}$ with respect to phantom thickness, with thicknesses ranging from 2.54 to 35.56. For this same fluoroscopic protocol, variation of the X‐ray tube potential with phantom thickness is shown in Fig. 14, variation of mA with phantom thickness is shown in Fig. 15, and variation of the copper filter thickness with phantom thickness is shown in Fig. 16.

## IV. DISCUSSION & CONCLUSIONS

### A. Air kerma rates (${\dot{K}}_{a,r}$)

All ${\dot{K}}_{a,r}$ values reported in this study were provided by the fluoroscope with a correction factor applied for accuracy that was determined by direct measurement with a calibrated ion chamber. The calibration factor was determined at a single beam quality with an ionization chamber that was calibrated for diagnostic energies. However, no calibration lab currently offers a calibration that covers the complete beam quality spectrum encountered on interventional fluoroscopes employing copper filtration. The uncertainty in the correction factor, based on energy dependence, is approximately ±3% as reported by the ionization chamber manufacturer. As previously stated, there are also slight geometric differences between the fluoroscopes that result in an approximately 5% or less variation in the ${\dot{K}}_{a,r}$ due to the plane of measurement.

As evidenced in Fig. 1, the radiation output capability for several of the current‐generation systems has provided for potentially higher acquisition ${\dot{K}}_{a,r}$, exceeding 2.5 Gy/min for some systems. These ${\dot{K}}_{a,r}$ values, under certain circumstances, may be necessary for anatomic or procedural visualization, especially for complex procedures in morbidly obese patients. However, great caution must be used when allowing ${\dot{K}}_{a,r}$ to reach these levels as tissue reactions could be triggered with only a few short acquisitions. There are currently no regulatory limits for the acquisition imaging mode; therefore, these ${\dot{K}}_{a,r}$ values are allowable. Fluoroscope operators and persons training those operators must understand that these substantial air kerma rates are possible and under what circumstances they may be realized.

As shown in Fig. 5, the low‐dose fluoroscopic mode of operation for all four of the newest‐generation fluoroscopes yielded substantially lower ${\dot{K}}_{a,r}$ than the previous‐generation unit evaluated. This is the imaging mode that fluoroscopes should default to, requiring operators to choose a higher dose rate mode, if needed. Figure 5 shows that system A has capped the ${\dot{K}}_{a,r}$ in this mode of operation at a level corresponding to 8.8 mGy/min (1 R/min) under standard testing geometry (i.e., 30 cm from the image receptor with lead in the beam, which differs from the geometry of Fig. 5). Although most vendors choose to cap the output in the low‐dose fluoroscopic mode at approximately 50% of the allowable 88 mGy/min US limit (i.e., 44 mGy/min at 30 cm from the image receptor), capping the output at 10% of the limit likely leads to operators prematurely transitioning to higher dose rate fluoroscopic imaging modes. In Figure 5, the maximum ${\dot{K}}_{a,r}$ on system A was reached at approximately 30 cm of PMMA, and in that imaging mode, additional amounts of attenuation reduced the dose to the image receptor instead of increasing the X‐ray output. If sufficient image quality can be achieved at further reduced image receptor doses, then a reduced image receptor dose should be employed throughout the curve to the maximum permissible exposure rate limit. Otherwise, allowing a reduced dose to the image receptor at such an artificially low cap may prematurely prompt an operator to choose a higher dose setting (with associated higher image receptor dose rates), defeating the benefit of the low‐dose mode.

### B. kVp modulation

In general, the current generation systems use higher kVps in the fluoroscopic and acquisition curves (2, 6, 10, 14) while maintaining copper filtration thickness, as compared to the previous generation system. This results in higher beam quality and a reduced skin entrance dose for a given patient‐fluoroscope geometry and image receptor dose, but this could also adversely affect image contrast. System A increases kVp and reaches a maximum value more quickly than most of the other systems; however, this system also uses lower amounts of copper filtration and does not dynamically change the filter thickness within a given protocol.

### C. Tube current (mA) modulation

The modulation of the X‐ray tube current is reported in different ways by the vendors. Time averaged mA is reported by the current generation system A in the acquisition mode of imaging and by systems A and D in the fluoroscopic mode. Inflection points can be seen in the mA curves where the copper filtration thicknesses change for the units that employ dynamic filters. In general, the mA curves tend toward higher values as phantom thickness increases, until a phantom thickness is used that drives the X‐ray tube to approach its power limitations. At that point, the ADRC must decrease the mA for the higher kVp values which is evident in all of the mA curves in Fig. 15 for the high‐dose fluoroscopic curve.

### D. Use of copper filters

The use of copper X‐ray beam filters was ubiquitous among the vendors evaluated, although all of the manufacturers have different approaches to how the filters are employed. Two vendors offer up to 0.9 mm of Cu filtration; however, for the testing performed, only one vendor employed that amount during fluoroscopic imaging. The use of 0.9 mm of Cu occurred at low kVps, suggesting that the greatest benefit is likely limited to pediatric FGI procedures. All but one system had a dynamic approach to changing the copper filtration within a given imaging protocol, whereas the other system used a static filtration thickness determined by the chosen imaging protocol. All of the current‐generation systems have transitioned to using Cu filters during acquisition imaging, something not typically seen on older‐generation systems. The inclusion of Cu allows for a substantial reduction in skin entrance dose during acquisitions, which is the imaging mode that delivers the highest fractional radiation dose for many FGI procedures.

### E. Fluoroscopic operational characteristics: a team approach

The overall approach to imaging protocol optimization and the logic determining when to transition to higher dose imaging modes must be considered so that the end user can be presented with a full spectrum of dose rates (and image quality) optimized to the clinical task. To achieve this goal, all parties involved must diligently work together. Clinicians must understand the complexity of state‐of‐the‐art equipment and that optimizing an imaging protocol necessitates identifying the lowest image quality possible to successfully complete a clinical task; high image quality is not always necessary and comes at the cost of radiation dose to the patient and occupational dose to the clinical team. Physicists must understand the vendor‐specific approaches to the imaging protocol parameters and the ways in which they can be manipulated to achieve a clinically optimized imaging protocol. Equipment vendors must be forthright regarding the parameters they use for image acquisition and processing and how those parameters may be modified to achieve a desired change in radiation dose rate or image quality, something currently lacking from most vendors.

## Supporting information

Supplementary Material

Supplementary Material

Supplementary Material

Supplementary Material

Supplementary Material

Supplementary Material

Supplementary Material

Supplementary Material

Supplementary Material

Supplementary Material

Supplementary Material

Supplementary Material

Supplementary Material

Supplementary Material

Supplementary Material

Supplementary Material

Supplementary Material

Supplementary Material

Supplementary Material

Supplementary Material

Supplementary Material

## Appendix A. Deviation due to Differences in Floor‐to‐Table Height

The approximately 5% geometric deviation indicated in the text was calculated using the data from the unit with the largest floor‐to‐table height distance; these data were then adjusted to represent that system at the same table height as the shortest floor‐to‐table distance (90 cm, from Table 2):

(A1) $$\text{Dev}={\left(\frac{\frac{{\text{SID}}_{\text{table new height}}}{{\text{SSD}}_{\text{table new height}}}}{\frac{{\text{SID}}_{\text{table initial height}}}{{\text{SSD}}_{\text{table nitial height}}}}\right)}^{2}={\left(\frac{\frac{(\text{New table height}‐\text{floor to focal spot dis})+\text{phantom thickness}}{\text{New table height}‐\text{floor to focal spot dis}}}{\frac{(\text{Initial table height}‐\text{floor to focal spot dis})+\text{phantom thickness}}{\text{Initial table height}‐\text{floor to focal spot dis}}}\right)}^{2}={\left(\frac{\frac{(90-31)+35.6}{\left(90-31\right)}}{\frac{63.5+35.6}{63.5}}\right)}^{2}={\left(\frac{1.6.3}{1.561}\right)}^{2}=1.05$$
