# Supplementary figures and images for: Approaches to interventional fluoroscopic dose curves
Source: J Appl Clin Med Phys. 2016 Jan 8;17(1):342–52. doi: 10.1120/jacmp.v17i1.5788 (PMC5690202; doi:10.1120/jacmp.v17i1.5788)

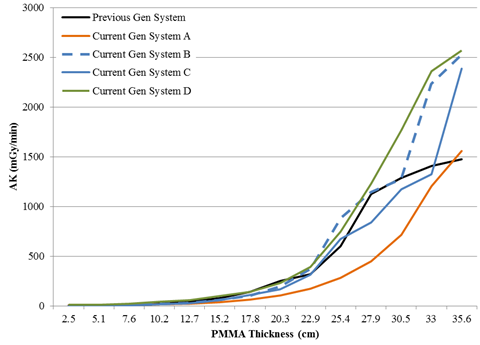

Supplement: Supplementary file 1 — Supplementary Material [file ACM2-17-342-s001.png]

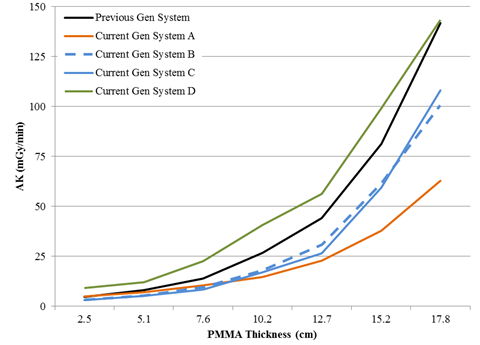

Supplement: Supplementary file 2 — Supplementary Material [file ACM2-17-342-s002.png]

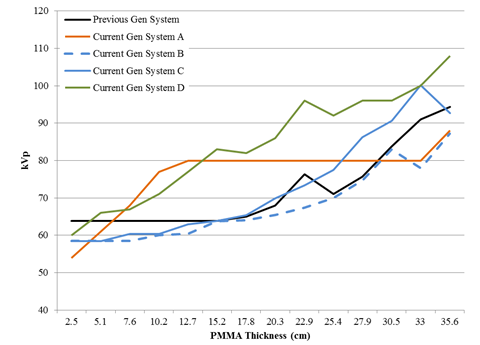

Supplement: Supplementary file 3 — Supplementary Material [file ACM2-17-342-s003.png]

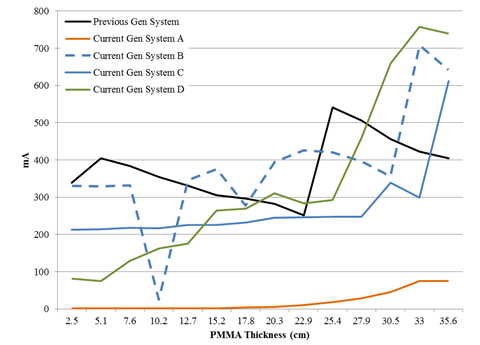

Supplement: Supplementary file 4 — Supplementary Material [file ACM2-17-342-s004.png]

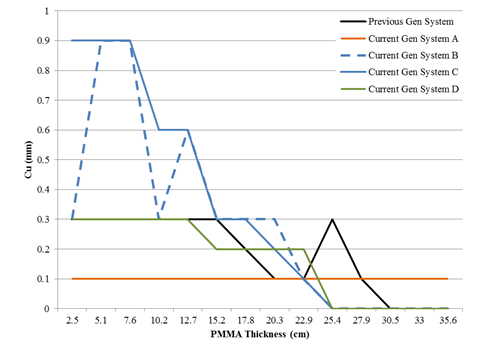

Supplement: Supplementary file 5 — Supplementary Material [file ACM2-17-342-s005.png]

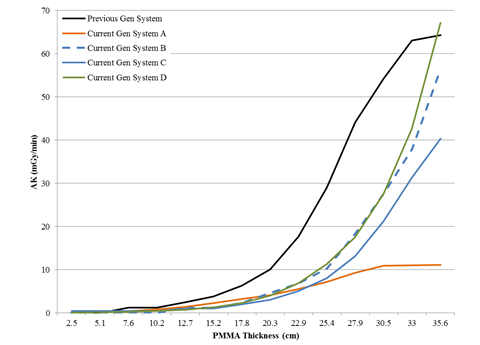

Supplement: Supplementary file 6 — Supplementary Material [file ACM2-17-342-s006.png]

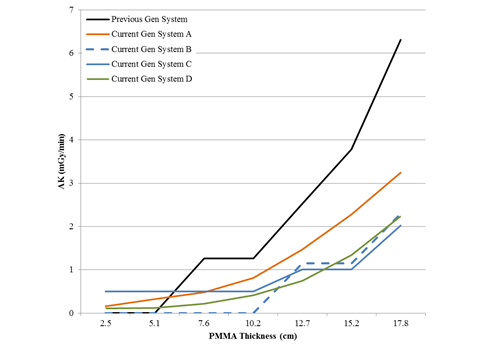

Supplement: Supplementary file 7 — Supplementary Material [file ACM2-17-342-s007.png]

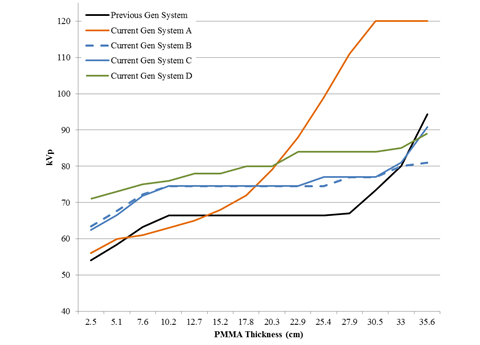

Supplement: Supplementary file 8 — Supplementary Material [file ACM2-17-342-s008.png]

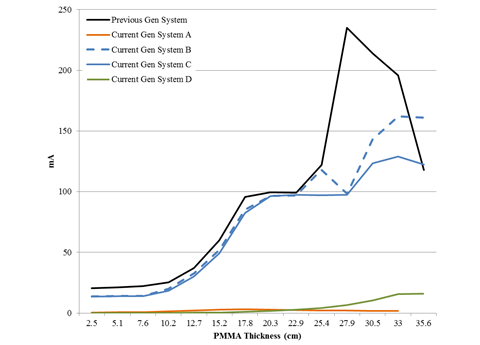

Supplement: Supplementary file 9 — Supplementary Material [file ACM2-17-342-s009.png]

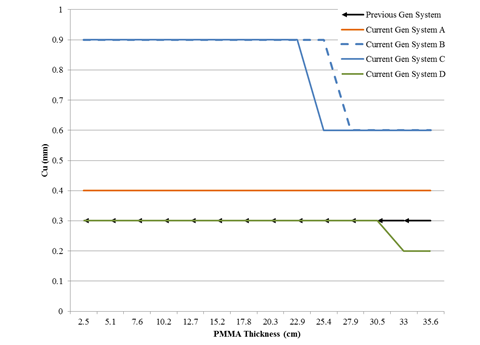

Supplement: Supplementary file 10 — Supplementary Material [file ACM2-17-342-s010.png]

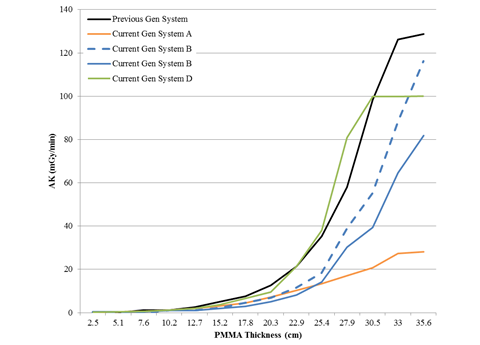

Supplement: Supplementary file 11 — Supplementary Material [file ACM2-17-342-s011.png]

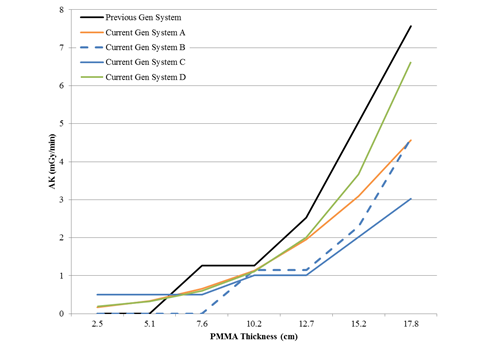

Supplement: Supplementary file 12 — Supplementary Material [file ACM2-17-342-s012.png]

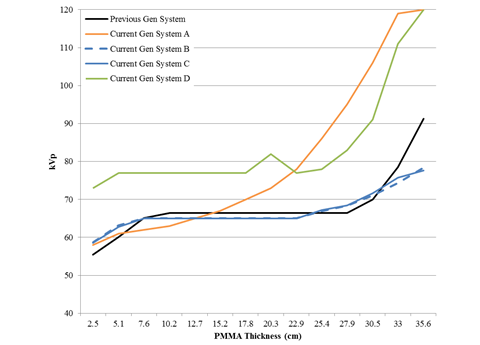

Supplement: Supplementary file 13 — Supplementary Material [file ACM2-17-342-s013.png]

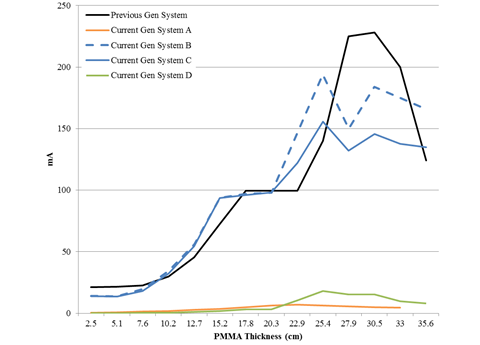

Supplement: Supplementary file 14 — Supplementary Material [file ACM2-17-342-s014.png]

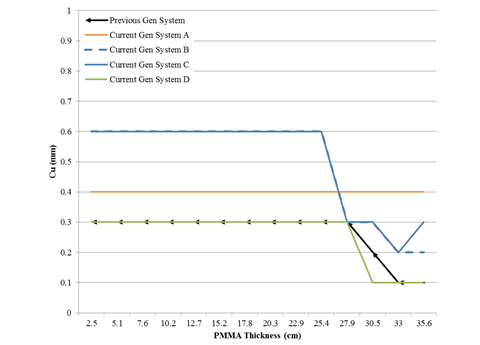

Supplement: Supplementary file 15 — Supplementary Material [file ACM2-17-342-s015.png]

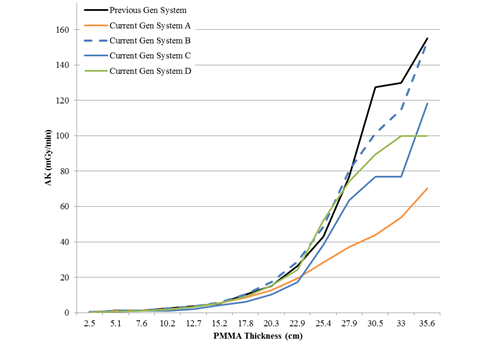

Supplement: Supplementary file 16 — Supplementary Material [file ACM2-17-342-s016.png]

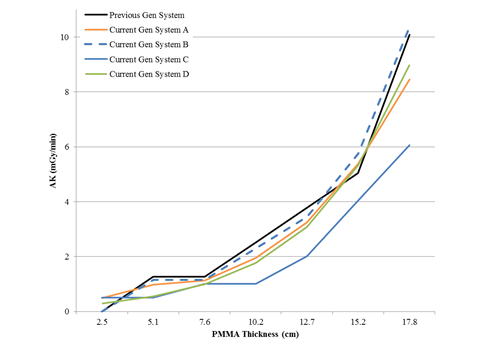

Supplement: Supplementary file 17 — Supplementary Material [file ACM2-17-342-s017.png]

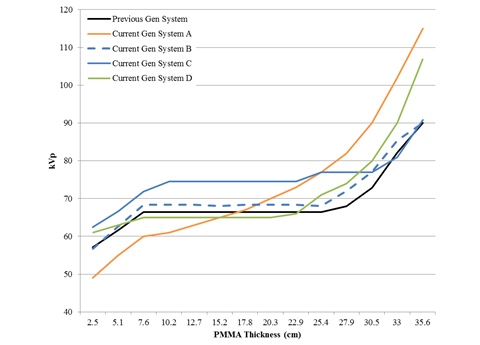

Supplement: Supplementary file 18 — Supplementary Material [file ACM2-17-342-s018.png]

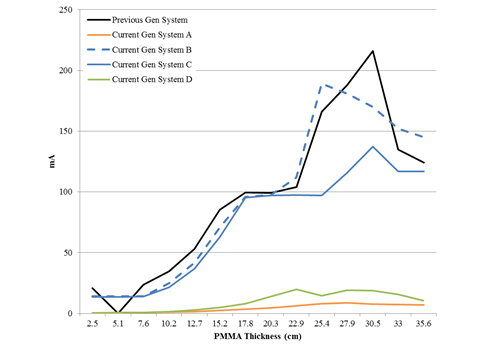

Supplement: Supplementary file 19 — Supplementary Material [file ACM2-17-342-s019.png]

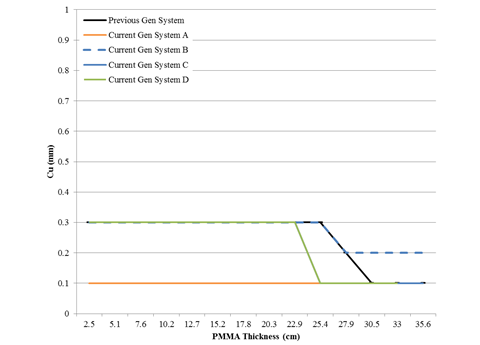

Supplement: Supplementary file 20 — Supplementary Material [file ACM2-17-342-s020.png]

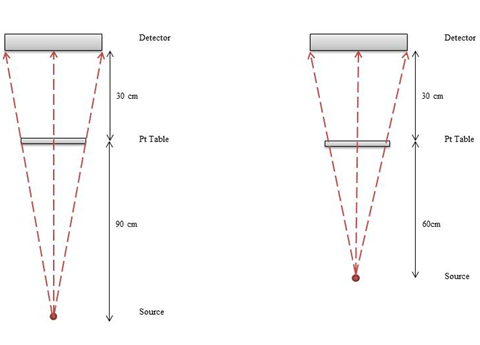

Supplement: Supplementary file 21 — Supplementary Material [file ACM2-17-342-s021.png]
